# Does depression moderate handwashing in children?

**DOI:** 10.1186/s12889-017-4638-4

**Published:** 2017-08-01

**Authors:** Jurgita Slekiene, Hans-Joachim Mosler

**Affiliations:** EAWAG, Swiss Federal Institute of Aquatic Science and Technology, Environmental Social Sciences, Environmental and Health Psychology, Überlandstrasse 133, P.O. Box 611, -8600 Dübendorf, CH Switzerland

**Keywords:** School children, Behavior change, Depression, Handwashing with soap and water, Diarrhea prevention, Peri-urban Zimbabwe

## Abstract

**Background:**

Although diarrheal diseases are preventable and treatable, they are the leading cause of child mortality and morbidity as a consequence of poor hygiene and contaminated water. Handwashing with soap is an effective method for preventing and decreasing the incidence of diarrhea. However, mental disorders such as depression can substantially moderate an individual’s ability to cope with daily life and can exert a negative influence on daily hygiene activities such as handwashing with soap, especially in children. The aim of this study was to explain the influence of depression on pupils’ hand-washing behavior in Zimbabwe.

**Methods:**

In a cross-sectional study, face-to-face interviews were carried out with primary school pupils in peri-urban Harare, Zimbabwe (*n* = 556) using a quantitative questionnaire to assess handwashing and its behavioral determinants in school settings. The Center for Epidemiological Studies Depression Scale for Children (CES-DC) was used to assess depression.

**Results:**

More than half of the assessed children were depressed. Self-reported handwashing with soap among depressed children was significantly lower than among non-depressed children. Almost all behavioral determinants of hand-washing behavior were significantly lower in depressed children. The behavioral determinants worked differently in the depressed children than in the non-depressed children’s group. The effects of important behavioral determinants on handwashing were moderated by depression.

**Conclusions:**

Depression exerts a negative influence on handwashing in children. These results suggest depression-relieving measures should be conducted together with any water, sanitation, and hygiene (WASH) interventions to make such interventions more effective.

## Background

Diarrheal diseases are one of the leading causes of morbidity and mortality worldwide [1]. Handwashing with soap is an effective method for preventing and decreasing diarrheal diseases by 42–47% [2, 3] and is cost effective [4]. The importance of handwashing with soap at critical times, before eating and after using the toilet, has been reported in previous empirical studies [5, 6]. However, although handwashing with soap is effective, rates of handwashing compliance are low [7].

There is evidence that internal mental conditions such as mental disorders may hinder daily activities. As handwashing is an activity that has to be performed many times in many situations during a day, this behavior might be especially prone to moderation by internal mental conditions. Depression seems to be a prevalent mental disorder, especially in developing countries [8]. According to the World Health Organisation (WHO) definition, depression can be long term or episodic, substantially impairing an individual’s ability to function at work or school and to cope with daily life [9].

Significant associations were identified between personal health-protective behaviors and psychological well-being and symptoms of depression by Allgower et al. [10]. Mental disorders, especially depression and posttraumatic stress disorder (PTSD), are common long-term psychological outcomes in emergency situations associated with conflict or natural disasters [11]. Other sources of negative mental health outcomes in children are violence and child abuse; the highest rates are in developing countries. About one in three girls is maltreated during childhood in Kenya, the Republic of Tanzania, Swaziland, and Zimbabwe. [12]. In Zimbabwe, the prevalence of mental health disorders, including depression, is very high [8, 13]. Some researchers have suggested that the universality of depression symptoms across cultures allows the same diagnostic methods for identifying sufferers to be used in Zimbabwe as elsewhere [8, 13].

If depression has a moderating effect on personal health behaviors such as handwashing, then the question that arises is how this moderating effect works. Hawkins and colleagues [14] suggested that behavioral variables had possible mediating effects on the relationship between symptoms of depression and self-reported health-related behaviors. Thus, the question becomes how depression moderates the behavioral determinants that influence handwashing behavior. It might be that these determinants can exert divergent influences on handwashing so that the values of behavioral determinants of handwashing are different in depression sufferers than in the non-depressive.

To understand how depression can influence handwashing, the relevant behavioral determinants must be identified. The Risks, Attitudes, Norms, Abilities, and Self-regulation (RANAS) model of behavior change provides the most comprehensive compilation of behavioral determinants in water, sanitation, and hygiene (WASH). The usefulness of this model in explaining handwashing has been demonstrated in a recent study in rural Ethiopia [15] and in an emergency situation during the humanitarian disaster in Haiti [16]. The five factor blocks of the RANAS model cover risk, attitude, norm, ability, and self-regulation factors [17]. Risk factors include factual knowledge about the transmission of a disease, prevention options, personal consequences, perceived vulnerability, and the perceived severity of contracting a disease. Attitude factors include beliefs about the costs and benefits of a particular behavior and feelings associated with the behavior. Norm factors, such as the behavior of others, others’ (dis)approval, and personal importance, relate to perceived social influence. Ability factors include people’s confidence in the performance of a particular behavior. Self-regulation factors include management of conflicting goals, distracting cues, and barriers to performing the behavior. Important determinants are commitment, perceived habit, and remembering the behavior.

This paper aims to explain the underlying mechanisms of children’s hand-washing behavior in Zimbabwe using the behavioral determinants of the RANAS model. The objective of our research was to investigate the possible negative influence of depression on hand-washing behavior among primary-school children in Harare. Working from the suggestions of existing research and considerations of mental health and depression, we address the following research questions in this paper: 1) Does handwashing frequency differ between depressed and non-depressed primary school children in Harare? 2) Is the influence of the behavioral determinants of handwashing weaker on depressed children than on non-depressed children? 3) Does the impact of the sum of behavioral determinants on handwashing in depressed children differ from that in non-depressed children? 4) Do different behavioral determinants influence handwashing in depressed and non-depressed children? 5) Does depression moderate the effects of behavioral determinants on handwashing?

## Methods

### Research area

The present study was conducted in primary schools in peri-urban and urban areas of Harare, the capital of the Republic of Zimbabwe. One of the World Development Indicators (WDI), the primary school enrollment rate, is surprisingly high in Zimbabwe: 97% [18]. Twenty primary schools in 11 suburbs of urban and peri-urban Harare were randomly selected for the present study.

#### Data collection

The data collection for the cross-sectional study was performed in Zimbabwe during 4 weeks in July and August 2014. One local field coordinator and eight data collectors, most of whom held a university degree in social sciences or a similar discipline, were recruited and employed full time. A 5-day interviewer training program was organized by the research team before the data-collecting tools were pre-tested in the field. During the training, the group learned about the theoretical background of questionnaires, ethics, and the aims of the survey, took part in role-play exercises focused on interviewing children, and practiced using tablet computers.

### Quantitative face-to-face interviews

Data collection took place in the 20 selected primary schools. The interviews with the children were usually carried out in the school computer room, classroom, or school staff room. The data collectors were always supervised in the field by at least one research team member and a local field coordinator. The children were randomly selected (30 per school), and written informed consent was obtained from the parents of all children included in the study before the research team entered the schools. Participation in the interview was voluntary, and we also obtained written assent from the children. Each interview lasted between 20 and 25 min, and response cards were used during the interview. The questionnaire was translated from English to Shona by a local translator and back-translated by another local translator to ensure accuracy.

The data were collected using tablets. The questionnaires were installed on the tablets using Excel, OpenDataKit, an open-source software package developed at the University of Washington’s Department of Computer Science and Engineering, and formhub.org, an online open-source tool from the Sustainable Engineering Lab at the Earth Institute, Columbia University.

### Sample

A total of 31,162 children were enrolled in the 20 selected primary schools. The number of children per school ranged from 933 to 2666. The final survey sample consisted of 556 children (50% female (*n =* 278). The age of the participants ranged between 6 and 14 years (M *=* 9.4; SD *=* 1.6). Within the sample, Grade 1 was attended by 4.7% (*n =* 26) of the children, Grade 2 by 12.4% (*n =* 69), Grade 3 by 24.9% (*n =* 138), Grade 4 by 21.4% (*n =* 119), Grade 5 by 19.5% (*n =* 108), Grade 6 by 16.6% (*n =* 92), Grade 7 by 0.5% (*n =* 3), and missing system 0.2% (*n =* 1).

### Questionnaires and measures

We assessed the determinants associated with handwashing, hand-washing behavior, and symptoms of depression in children. First, using the RANAS behavior change model, we aimed to detect the behavioral determinants that explain hand-washing behavior in children. Second, we operationalized the target behavior as handwashing with soap and water at critical times (after using the toilet and before eating).

### Handwashing and behavioral determinants

Using a questionnaire for adults that was built on the RANAS model and has been used successfully in previous research studies in developing countries as a basis [15, 19, 20], we developed a quantitative questionnaire for children. Using the new questionnaire, we aimed to assess self-reported handwashing with soap at critical times and to identify the behavioral determinants that explain handwashing among primary school children. We chose this approach because, although self-reports are prone to reporting bias, they have been found to be associated with child diarrhea [5].

When defining the target population, we considered Piaget’s observations and conclusions that a child’s mind is based on logical, inductive, flexible, practical, and organized thinking from the age of approximately 7 years [21, 22]. Response cards using a 4-point rating scale were created and presented to the pupils during the interviews. Using response cards helps to increase children’s motivation to respond, to improve instructional management, and to make the interview more interesting [23–25]. The response cards were designed and produced in collaboration with marketing professionals. The card color, sky blue, was chosen for its linguistic and cultural connotations.

### Depression

To identify the groups of children who are at risk for developing depression in primary schools in Harare, we used the Center for Epidemiological Studies Depression Scale for Children (CES-DC) [26, 27], a reliable and valid depression screening instrument (internal consistency *α* = .74–.89; effect size = .72; sensitivity = 80). The CES-DC uses a 20-item rating scale ranging from 0 to 3. The recommended cutoff point for an initial validation study was a score of ≥15 (score range 0–60) [28]. The CES-DC was translated from English to Shona by a local translator and back-translated by another local translator to ensure accuracy. To adapt the questionnaire to the interview setting, some changes were needed, such as converting the item text from the first person (“I”) to second person (“You”), and inserting the same response scales as in the quantitative questionnaire for children based on the RANAS model (we replaced “some” with “a medium amount” and “a lot” with “a great deal”).

### Statistical analysis

The statistical analysis was conducted with IBM SPSS 22 Statistics software. Frequencies, descriptives, zero-order correlation, and linear regression analysis methods were applied. Additionally, a new linear regression analysis method for SPSS, PROCESS (macro for SPSS) [29, 30, 31], was used to examine our last research question concerning depression as a moderator of hand-washing behavior. The PROCESS analysis method not only identifies the moderation effect but also, if the interaction is significant, identifies the complexity of the moderation using simple slopes analysis to detect conditional effects of the predictor on the outcome with low, average, and high moderator values (low = 1 SD below the mean, average = the mean, high = 1 SD above the mean). Finally, the Johnson–Neyman technique makes it possible to explore the significance region’s conditional predictor effects on the outcome at the moderator values, where the predictor no longer predicts the outcome [30].

## Results

### Prevalence of depression

**Fig. 1:**
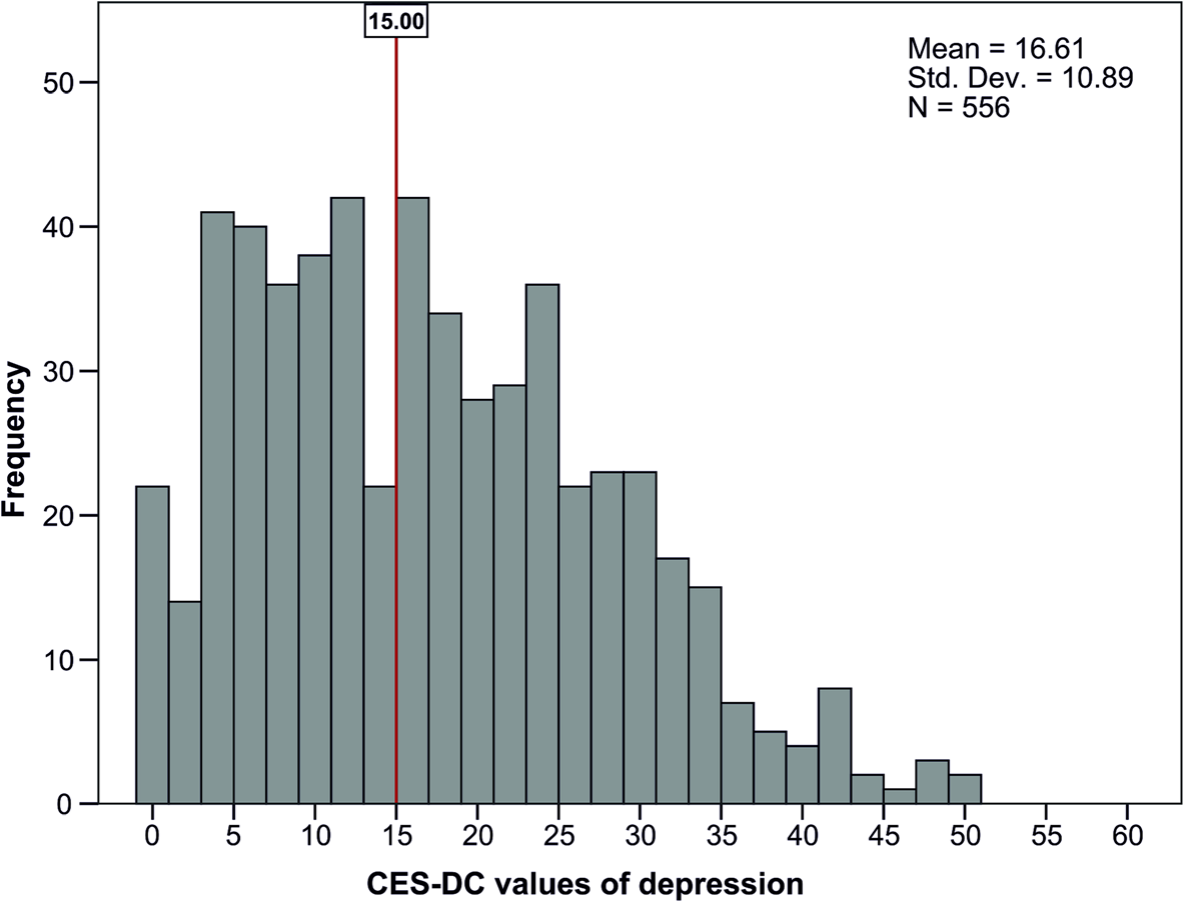
Prevalence of depression among primary school children in Harare (CES-DC score range, 0–60; cutoff point score, ≥ 15)

In our sample of 556 children, we identified 301 (54%) children with a score equal to or above 15 on the CES-DC scale (M *=* 16.6, SD *=* 10.9, CES-DC cutoff point ≥15), as shown in Fig. 1. That is, more than half of the children were depressed.

#### Influence of depression on handwashing and behavioral determinants

We asked, first, whether handwashing frequency differs between depressed and non-depressed among primary school children in Harare. We compared the groups based on scores for symptoms of depression, which was assessed with the CES-DC (cutoff point ≥15, scale range 0–60). There is a significant negative relationship between depression and self-reported handwashing (*p* = 0.01, *r* = −.112). The simple linear regression analysis demonstrated that depression is a significant predictor of handwashing at school (adj. *R* ^*2*^ = .013, *β* = −.112, *p* = 0.01). That is, if children are depressed, they wash their hands less often. The mean of self-reported handwashing with soap at school in depressed children (M *=* 2.57; SD *=* 0.99) was lower than in non-depressed children (M *=* 2.81; SD *=* 1.14). The *t*-test revealed that the differences in self-reported handwashing between depressed and non-depressed children were statistically significant (*p* = .009) and that the related effect size (*d* = .23) is small [32].

The analysis of the second research question, whether the influence of the behavioral determinants of handwashing is weaker on depressed children than on non-depressed children, revealed that nearly all of the behavioral determinants were significantly lower in depressed children than in non-depressed children. Table 1 shows the means and standard deviations for depressed and non-depressed children. The largest differences were found in perceived vulnerability (fear of contracting diarrhea), perceived severity (personal consternation in case of contracting diarrhea), health knowledge, instrumental beliefs (benefits and costs of handwashing), affective beliefs (experiencing pleasure during handwashing), injunctive norm (what others think about handwashing), remembering (forgetting), commitment (importance of handwashing and guilt in case of not washing hands at critical times), and intention to always wash hands with soap. The related population effect sizes (from *d* = .20 to *d* = .50) lie in a range between small and medium [32].

**Table 1:** Means, standard deviations, and differences between means of determinants of handwashing (depressed vs. non-depressed children)

Behavioral determinants	Depresse *N =* 291	Non-depressed *N =* 238	Differences between groups	
			*Sig. (2-tailed)*	*Cohen’s*
	M (SD)	M (SD)	p	d
Risk factors				
Perceived vulnerability	2.66 (1.26)	3.03 (1.21)	.0001	0.30
Perceived severity	2.74 (1.26)	3.23 (1.17)	.0001	0.40
Health Knowledge	0.20 (0.13)	0.24 (0.13)	.0001	0.30
Attitude factors				
Instrumental beliefs	1.84 (0.65)	1.64 (0.61)	.0001	0.32
Affective beliefs-liking	3.63 (0.72)	3.83 (0.53)	.0001	0.32
Affective beliefs-disgust	2.82 (1.01)	3.02 (1.08)	.020	0.20
Norm factors				
Descriptive norm	2.30 (0.96)	2.40 (1.09)	*ns*	–
Injunctive norm	3.27 (0.90)	3.57 (0.83)	.0001	0.35
Ability factors				
Action knowledge	0.79 (0.31)	0.83 (0.29)	*ns*	–
Action self-efficacy	3.09 (0.96)	3.15 (1.07)	*ns*	–
Maintenance self-efficacy	2.79 (1.02)	3.04 (1.11)	.006	0.24
Self-regulation factors				
Action control	3.18 (0.90)	3.41 (0.82)	.001	0.27
Remembering	3.17 (0.82)	3.45 (0.76)	.0001	0.35
Commitment	3.11 (0.63)	3.34 (0.62)	.0001	0.37
Additional factors				
Habit	2.79 (0.94)	2.95 (1.04)	*ns*	–
Intention	3.24 (0.81)	3.59 (0.68)	.00001	0.47

Scale ranges = 1 (not at all) - 4 (great deal). Scale ranges by categorical variables health and action knowledge = 0.0, 0.2, 0.4, 0.6, 0.8, 1.0. Diff. between groups: *t*-test calculation for equality of means

The third research question, whether the impact of the sum of behavioral determinants on handwashing in depressed children differs from that in non-depressed children*,* was tested using the multiple linear regression analysis with self-reported handwashing as the dependent variable and behavioral determinants as predictors. All RANAS factors and the additional factor of habit were included in the analysis.

The regression analysis shown in Table 2 demonstrated an adjusted *R* ^*2*^ value of .359 in the depressed children group; that is, the behavioral factors explained 36% of the variance of self-reported handwashing. In the non-depressed children group, the calculated adjusted *R* ^*2*^ value of .471 was higher than in the depressed children group; that is, the behavior factors explained 47% of the variance of self-reported handwashing. The related population effect size of *f* ^*2*^ is large (*f* ^*2*^ *≥* .35) [32].

**Table 2:** Regression of RANAS factors by CES-DC levels of symptoms of depression in children

Behavioral determinants	Self-reported handwashing with soap	
	Depressed	Non-depressed
	*β*	*β*
Risk factors		
Perceived vulnerability	.065	.032
Perceived severity	−.104*	.011
Health knowledge	.003	.070
Attitude factors		
Instrumental beliefs	.104*	.006
Affective beliefs- liking	−.088	.033
Affective beliefs- disgust	−.069	.009
Norm factors		
Descriptive norm	.186**	.096
Injunctive norm	.105	−.065
Ability factors		
Action knowledge	.089	.073
Action self-efficacy	.243***	.469***
Maintenance self-efficacy	.097	.107
Self-regulation factors		
Action control	.042	−.006
Remembering	.153*	.068
Commitment	−.088	−.070
Habit	.204***	.208***

*p ≤ 0.05; **p ≤ 0.01; ***p ≤ 0.001. Scale range for variable depression: Yes = 1, No = 0 (CES-DC cutoff point *≥15). β* = standardized beta values*.* Adj. R^2^ in depressed children group = .359 (*N* = 291); Adj. R^2^ in non-depressed children group = .471 (*N* = 238)

To evaluate the fourth research question*,* whether different behavioral determinants influence handwashing in depressed children and non-depressed children, we conducted a multiple linear regression analysis with handwashing as the outcome and all behavioral determinants as predictors (see Table 2 above). In depressed children, significant predictors of handwashing were the perception of severity when contracting diarrhea (perceived severity), the expected results of costs and benefits (instrumental beliefs), social norms, such as the behavior of other people (descriptive norm), belief in the ability to organize and execute certain behaviors (action self-efficacy), remembering, and routinized behaviors (habits). In contrast, in non-depressed children, we identified two behavioral determinants associated with handwashing: the belief in the ability to organize and execute handwashing (action self-efficacy) and routinized performance of a behavior (habit). The influence of action self-efficacy was two times stronger in non-depressed children than in depressed children.

### Depression as moderator of handwashing

For the last research question, whether depression moderates the effects of behavioral determinants on handwashing, we tested a moderations model. Within this model, each factor was tested separately as a predictor of handwashing with depression as a moderator: behavioral factor as a predictor, CES-DC scores as a moderator, and self-reported hand-washing behavior as an outcome. Behavioral determinants that were moderated in depressed children were health knowledge, instrumental beliefs, affective beliefs, and commitment (Table 3). Depression exerts not only direct but also in some cases indirect negative effects on children’s handwashing.

**Table 3:** Interaction effects between depression and behavioral factors on self-reported handwashing

Interactions with depression	*b,* 95% CL	*t*	Levels of the moderator (CES-DC Scores)	CES-DC Scores demarcating significance region (% Below/Above)
Health knowledge	−0.080* [−0.144, −0.015]	−2.43	low, average	4.80 (score 21) (67*%*, 32*%*)
Instrumental beliefs	0.019** [0.007, 0.031]	3.10	low	−3.16 (score 13) (44*%*, 56*%*)
Affective beliefs- like	−0.013* [−0.023, −0.002]	−2.40	low, average, high	13.44 (score 30) (88*%*, 12*%*)
Commitment	−0.016* [−0.028, −0.003]	−2.45	low, average, high	11.65 (score 28) (84*%*, 16*%*)

*p ≤ 0.05, **p ≤ 0.01. *N* = 556, confidence intervals = 95%. Levels of moderator calculated with simple slopes analysis: MZ ± 1 SD (low = MZ - 1 SD, average = MZ, high = MZ + 1 SD). Significance region: conditional effect of factor on handwashing at values of CES-DC (scale 0–60)

A significant interaction effect was found between depression and health knowledge, which represents the child’s knowledge about the possibilities of avoiding diarrheal diseases (*b = −*0.080, 95% CI [−0.144, −0.015], *t = −*2.43*, p ≤* 0.01). This result implies that the effects of knowledge (the more the children know about health issues, the more they wash their hands) on handwashing was moderated by depression in children with high CES-DC scores. The analysis showed that in 31% of the children with a depression score equal to or above 21, health knowledge had no effect on handwashing.

A significant interaction between depression and instrumental beliefs, which represents the perceived costs and benefits of handwashing in a child’s mind (*b =* 0.019, 95% CI [0.007, 0.031], *t =* 3.10, *p ≤* 0.01), indicates that the relationship was moderated by depression. In children with high and average CES-DC scores, instrumental beliefs had no significant effect on handwashing. Instrumental beliefs had no significant effect on handwashing among more than half of the participants (56%, score equal to or above 13 on the CES-DC scale).

Further, the interaction effect between depression and affective beliefs, representing an individual’s feelings during handwashing with soap, was significant as well (*b = −*0.013, 95% CI [−0.023, −0.002], *t = −*2.40*, p ≤* 0.05). Affective beliefs had no effect in 12% of the assessed children (score equal to or above 30 on the CES-DC depression scale).

The interaction effect between commitment, which represents the importance of handwashing and guilt when handwashing is not performed, and depression was significant: *b = −*0.016, 95% CI – 0.028, −0.003], *t = −*2.45*, p ≤* 0*.*05*.* In children with high CES-DC scores, the effect of commitment on handwashing was moderated by depression. The significance region was delimited by a score of 28 on the depression scale, meaning that in 16% of the children, commitment had no effect on hand-washing behavior.

The moderation analysis revealed more significant interactions, for example, between perceived severity, action self-efficacy, maintenance self-efficacy, action control, remembering, habit, and intention and depression. According to our analysis, the interaction effects were very weak.

## Discussion

### Interpretation of results

The purpose of the present study was to examine the influence of depression on handwashing and hand-washing determinants. We aimed to understand the effects of depression in children on hand-washing behavior through the underlying behavioral determinants.

More than half of the children assessed in our study were depressed, which is in line with the findings of other studies in Zimbabwe [8, 13]. In line with our expectations, depressed children washed their hands less often. Our study showed that depression may moderate handwashing among school children; in general, depression exerts a negative influence on hand-washing behavior.

Almost all behavioral determinants were significantly weaker in depressed children than in non-depressed children. These differences could be attributed to altered mental states or affect in depressed children. The depressed children indicated that they more frequently forget to wash their hands, experienced less pleasure, and felt less guilty when they did not wash their hands, and their intention to wash their hands with soap was lower. They perceived themselves as less vulnerable to contracting diarrhea, and they were less aware of the severity of diseases. Furthermore, the depressed children cared less about what others thought when they did not wash their hands than non-depressed children. Our findings are in line with the definition of depression [9] that depression impairs an individual’s ability to function at work or school and to cope with daily life.

We established that the behavioral determinants we examined provide a better explanation of hand-washing behavior at critical times in non-depressed children than in depressed children. The study results revealed different hand-washing determinants in non-depressed children and depressed children. The strongest behavioral determinants among all children were action self-efficacy and habit. Action self-efficacy exerted an influence on handwashing that was twice as strong in non-depressed children as in depressed children. These results indicate that confidence in the ability to organize and execute the hand-washing behavior exerts less influence in depressed children. Our findings are in line with the prediction of Bandura’s theory of self-efficacy [33] that the development of individual self-efficacy beliefs is based on a range of information sources, including the emotional state of a person, and that higher levels of self-efficacy are associated with positive affect. Study results also indicate that awareness of the perceived severity of a disease exerts less influence on handwashing in depressed children. Further, the subjective feeling of being a better person may be a determinant of handwashing, because depression usually reduces that feeling; for example, depressed individuals usually hold negative thoughts about themselves. Depressed children also rely more on external stimuli than non-depressed children, for example, on descriptive norms, which represent others’ behavior, and remembering to wash their hands. More generally, the present findings are in line with previous research that the affective state of individuals can influence decisions or intentions to perform or not perform a certain behavior [34].

Finally, the results of moderation analysis imply that depression moderates the influence of several behavioral determinants of handwashing, such as health knowledge, instrumental belief, affective beliefs, and commitment. These findings imply, in line with Beck [35–37], that depression moderates the relationship between several behavioral determinants and handwashing through negative thought patterns or evaluations of oneself, of the environment, and of the future, thoughts of worthlessness, and thoughts of death and/or suicide. For instance, these could be thoughts concerning instrumental beliefs, such as “everything is bad around me, I can’t expect something good and it is hard and time-consuming, so why should I wash my hands?”

To our best knowledge, this study is the first empirical contribution to examine depression as a moderator of the relationship between behavioral determinants and handwashing.

### Practical implications

Natural disasters and conflicts are emergency situations that require immediate humanitarian and medical help, psychological care, and hygiene interventions. The psychological outcomes in emergency situations include depression and traumatic disorders (e.g., PTSD). Our findings regarding the negative influence of depression on hygiene behavior suggest a need for the integration of screening and treatment for mental disorders during or before a hygiene intervention. In particular, interventions to mitigate depression should focus on feelings within individuals aiming to increase positive emotions.

There is evidence that physical exercise has a positive effect on the mental well-being of a person, and exercise has been successfully applied in the treatment of anxiety and affective disorders such as depression [38]. An effective sports program could include squats, jumping, running around a school building, or physical exercises created specifically for the children’s cultural background.

## Study limitations

There are a few limitations to this study. First, the lack of soap in most primary schools in Harare (soap was observed in only 22% of the schools selected) impeded the identification of contextual determinants and direct observations of handwashing with soap. Second, no general physical examination of children was undertaken to rule out anemia, stigmata of nutritional deficiency and helminthic or worm infestations. Children were not screened for other comorbid psychiatric conditions, such as emotional and behavioral disorders (e.g., Attention-deficit/hyperactivity disorders (ADHD)), either. Our personal observations revealed withdrawn behavior and poor personal hygiene in interviewed children, but we do not have quantitative data on this. Further studies could include additional screening. Finally, our interpretations of the results rely on the cognitive theory of depression [36, 37]. According to this theory, depressed people show biased negative thinking and non-depressed people are more realistic. However, research on ‘depressive realism’ suggests a different view [39]: “*non-depressed people show illusory cognitions that seem to bolster psychological well-being; and (b) depressed people may be more realistic than their non-depressed counterparts, reflecting ‘depressive realism’”.*

## Conclusions

Individuals’ daily behaviors are associated with mechanisms underlying the relationship between emotion and cognition in its different dimensions, such as risk perception, knowledge, attitudes, memory, thinking, beliefs, and self-regulation. The present study confirmed that the strength of important hand-washing determinants among primary school children depends on the level of depression. Generally, depression exerts a negative influence on handwashing in children.

The findings of this research are preliminary and should be confirmed in other countries and contexts. Research on this topic is especially relevant to emergency situations, because it implies that depression-relieving measures conducted before or together with WASH interventions should make such interventions more effective. The effectiveness of various depression-mitigating strategies should be explored in field experiments. Additionally, measures should be developed to tailor such strategies so that they can be applied only to the individuals who need them.

To be maximally effective, behavior change campaigns must take the current findings into account. If we want to prevent and reduce diarrhea in developing countries, we need to develop effective evidence-based behavior change interventions; these should not only target behavioral determinants of handwashing but also integrate interventions for increasing positive emotions and decreasing symptoms of depression in children.

## Abbreviations

ADHD: Attention-deficit/hyperactivity disorders
CES-DC: The Center for Epidemiological Studies Depression Scale for Children
PTSD: Posttraumatic Stress Dissorder
RANAS: The Risks, Attitudes, Norms, Abilities, and Self-regulation
WASH: Water, Sanitation, Hygiene
WDI: World Development Indicators
WHO: World Health Organisation
